# Temperature and prey density drive growth and otolith formation of the world's most valuable fish stock

**DOI:** 10.1038/s41598-023-43168-w

**Published:** 2023-09-25

**Authors:** Claudia Ofelio, Marta Moyano, Michael Sswat, Fanny Rioual, Fabien Moullec, Arturo Aguirre-Velarde, Myron A. Peck

**Affiliations:** 1 Institute of Marine Ecosystem and Fishery Science, University of Hamburg, Große Elbstraße 133, 22767 Hamburg, Germany; 2 Valencian International University, C. del Pintor Sorolla, 21, 46002 Valencia, Spain; 3 Ocean and Earth Science, National Oceanography Centre, University of Southampton, Southampton, United Kingdom; 4 Norwegian Institute for Water Research (NIVA), Økernveien 94, 0579 Oslo, Norway; 5 Centre for Coastal Research, University of Agder, Postbox 422, 4604 Kristiansand, Norway; 6 GEOMAR Helmholtz Centre for Ocean Research Kiel, Düsternbrooker Weg 20, 24105 Kiel, Germany; 7 Laboratory of Environmental Marine Sciences (LEMAR), UMR 6539 (UBO/CNRS/IRD/Ifremer), Technopôle Brest-Iroise, Plouzané, France; 8 Laboratorio de Ecofisiología Acuática, Instituto del Mar del Perú (IMARPE), Esquina Gamarra y General Valle S/N Chucuito, Callao, Peru; 9 Department of Coastal Systems, Royal Netherlands Institute for Sea Research, P.O. Box 59, 1790 AB Den Burg, Texel, The Netherlands; 10 Marine Animal Ecology Group, Department of Animal Sciences, Wageningen University, Wageningen, The Netherlands

**Keywords:** Phenology, Marine biology

## Abstract

Peruvian anchovy (*Engraulis ringens*) represents the largest single-species fishery worldwide. Knowledge on how temperature and prey availability influences growth and age estimation during marine fish early life stages is critical for predicting bottom-up processes impacting stock productivity under changing environmental conditions. We reared Peruvian anchovy larvae at two temperatures (14.5 and 18.5 °C) and prey concentrations [high (HF), and low (LF)] from 6 to 30 days post-hatch (dph) to measure growth rate and examine daily deposition of otolith increments. Peruvian anchovy larvae grew faster at 18.5 °C compared to 14.5 °C. Larvae reared at low prey concentration (18.5-LF) and low temperature (14.5-HF) grew 61 and 35% slower, respectively, than those at high prey and warm temperature (18.5-HF). Age and growth rates of larvae were well depicted in the otolith microstructure of well-fed larvae at 18.5 °C. However, larvae reared at 18.5-LF or 14.5-HF, had only 55 and 49% of the expected number of daily otolith increments. Our results suggest caution when attempting to explore how ocean processes regulate small pelagic stocks, the productivity of which are largely driven by changes in the survival and growth of young larvae.

## Introduction

Peruvian anchovy, *Engraulis ringens*, represents the largest mono-specific fishery worldwide^1^ and is considered as the most abundant pelagic fish inhabiting the Southeast Pacific, playing a key intermediate role in the trophic network^2,3^. The large size of the Peruvian anchovy stock is linked to the high productivity and trophic transfer efficiency of the Humboldt Current System (HCS), which extends from Northern Peru to Southern Chile (4–36°S)^4^. These nutrient-rich waters support the rates of primary and secondary (i.e., zooplankton) production needed to fuel high individual and population growth rates of small pelagic fish^3^, with biophysical processes creating optimal environmental windows for the larval survival and growth of Peruvian anchovy^5,6^. The productivity of Peruvian anchovy has been associated with periods of high zooplankton biomass and strong upwelling^7^. Furthermore, anchovy normally inhabit waters within a relatively narrow (4 to 6 °C) range in temperatures^4,8^. The reliance of anchovy on specific feeding and temperature conditions helps explain the marked variability associated with both short- (i.e., annual) and long-term (i.e., decadal) ocean-climate variability associated with the El Niño Southern Oscillation (ENSO) and positive Pacific Decadal Oscillation (PDO) index, respectively^9^. Future climate projections suggest an increase in the frequency of ENSO events and zonal shifts in upwelling strength, both of which are expected to cause further shifts in the productivity of anchovy stocks along the Peruvian and Chilean coastal waters^10^.

The survival of fish larvae typically constitutes a bottleneck for the productivity of marine fish populations^11^. Getting reliable estimates on the effects of environmental variables on growth and survival of larvae and other early life stages of fish is essential for fisheries management, with water temperature and prey availability being two of the most important factors^12,13^. Both environmental drivers are closely related as warming directly influences larval feeding rates (through increased metabolic demands), but can also cause changes in prey communities^14^. The size of small pelagic fish stocks (i.e., sardines, anchovies, herrings or mackerels) can, thus, change rapidly when temperatures and prey fields become more or less suitable^15,16^. To better project future changes in valuable fisheries, it is important to gain a mechanistic understanding of how temperature and prey (among other factors) impact the vital rates (survival and growth) of early larval stages. In this sense, otoliths can serve as a “black box” to study vital rates in the wild^17^, but it is crucial to understand how growth is reflected in otoliths of larvae experiencing different temperatures and prey levels^18^.

The analysis of otolith microstructure (i.e., the number and width of daily increments) is a widely used technique in fisheries science to provide estimates of age and back-calculated somatic growth rate^19,20^. The technique has been widely applied to obtain the information needed for the stock assessment and management of the Peruvian anchovy stock in the HCS^21,22^. An underlying assumption is the daily formation of otolith micro-increments^23^ which should be validated to ensure that otoliths provide a robust estimate of fish age. This validation is generally performed under controlled conditions in the laboratory to minimize potentially confounding effects from other environmental factors.

The Peruvian anchovy, due to their high sensitivity to bottom-up forcing and to El Niño driven changes in habitat suitability, represents an ideal case study to investigate the interacting effect of temperature and prey density on larval growth and survival. Moreover, understanding how temperature and prey may affect growth as depicted in otoliths is needed to buttress science-based advice on the processes controlling survival and recruitment strength. Although the deposition of the first ring in the larvae of most anchovy species has been associated with the onset of the exogenous feeding, yolk sac absorption or eye pigmentation^24,25^, otolith development (e.g., the frequency of increment formation and increment widths) and somatic growth can be decoupled when feeding and growth conditions are suboptimal^26,27^. Research has started to examine the growth and development of Peruvian anchovy larvae under controlled (e.g. constant temperature and feeding) conditions in the laboratory^28^. The present study is the first to investigate how temperature (14.5 and 18.5 °C) and feeding conditions (low or ad libitum) impact on otolith formation of anchovy through the first month of life. Our results suggest caution when using otoliths to infer characteristics of survivors and identify processes controlling the recruitment dynamics of this stock.

## Results

### Growth traits and otolith microstructure analysis

**Figure 1:**
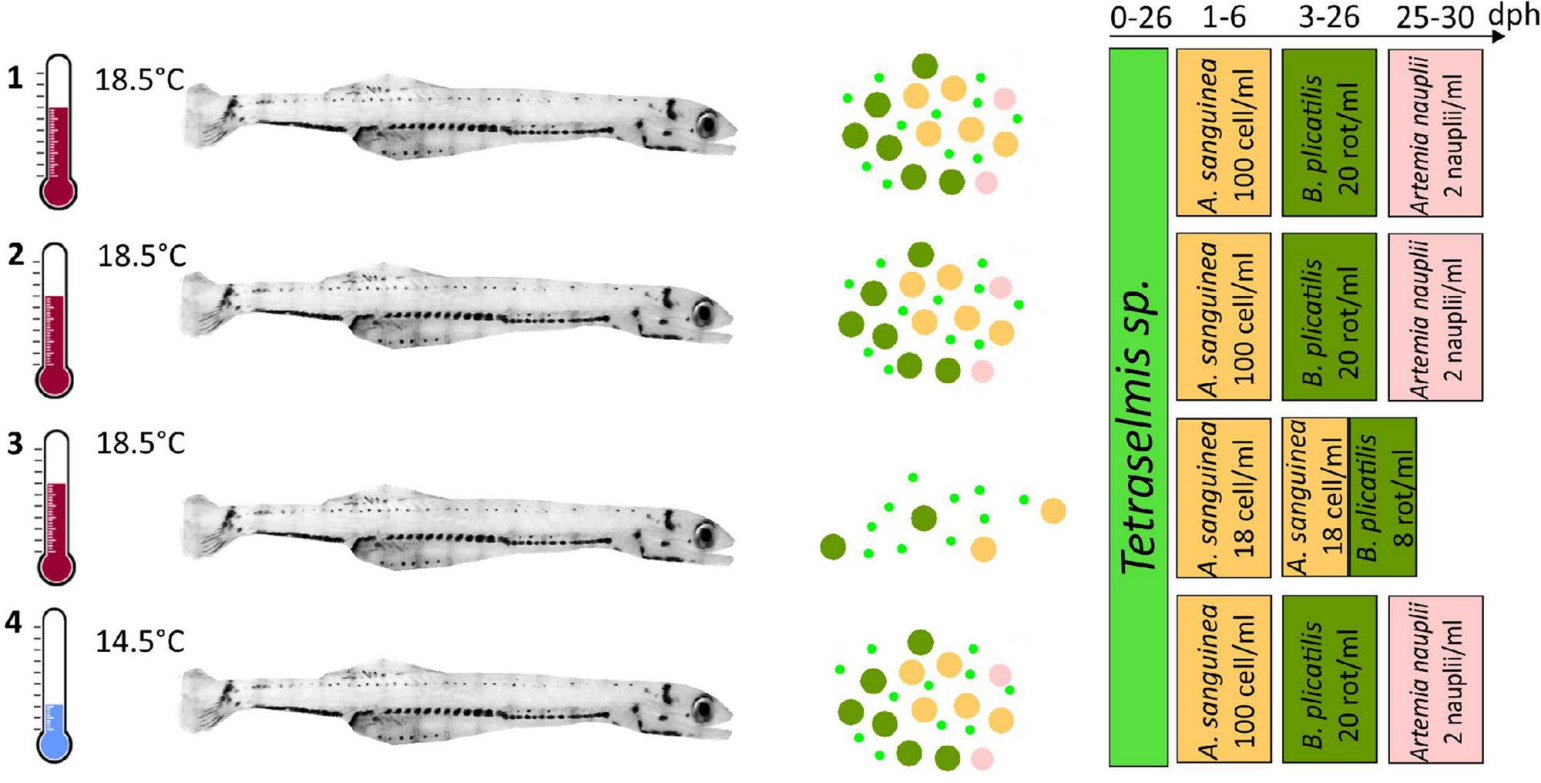
Experimental design of the Peruvian anchovy (*Engraulis ringens*) trials conducted in this study under laboratory conditions. (1): 18.5 °C, high food, batch-1; (2):18.5 °C, high food, batch-2; (3): 18.5 °C, low food, batch-3; (4): 14.5 °C, high food, batch-4. See methods for further details about the feeding plan. dph, days post-hatch.

**Figure 2:**
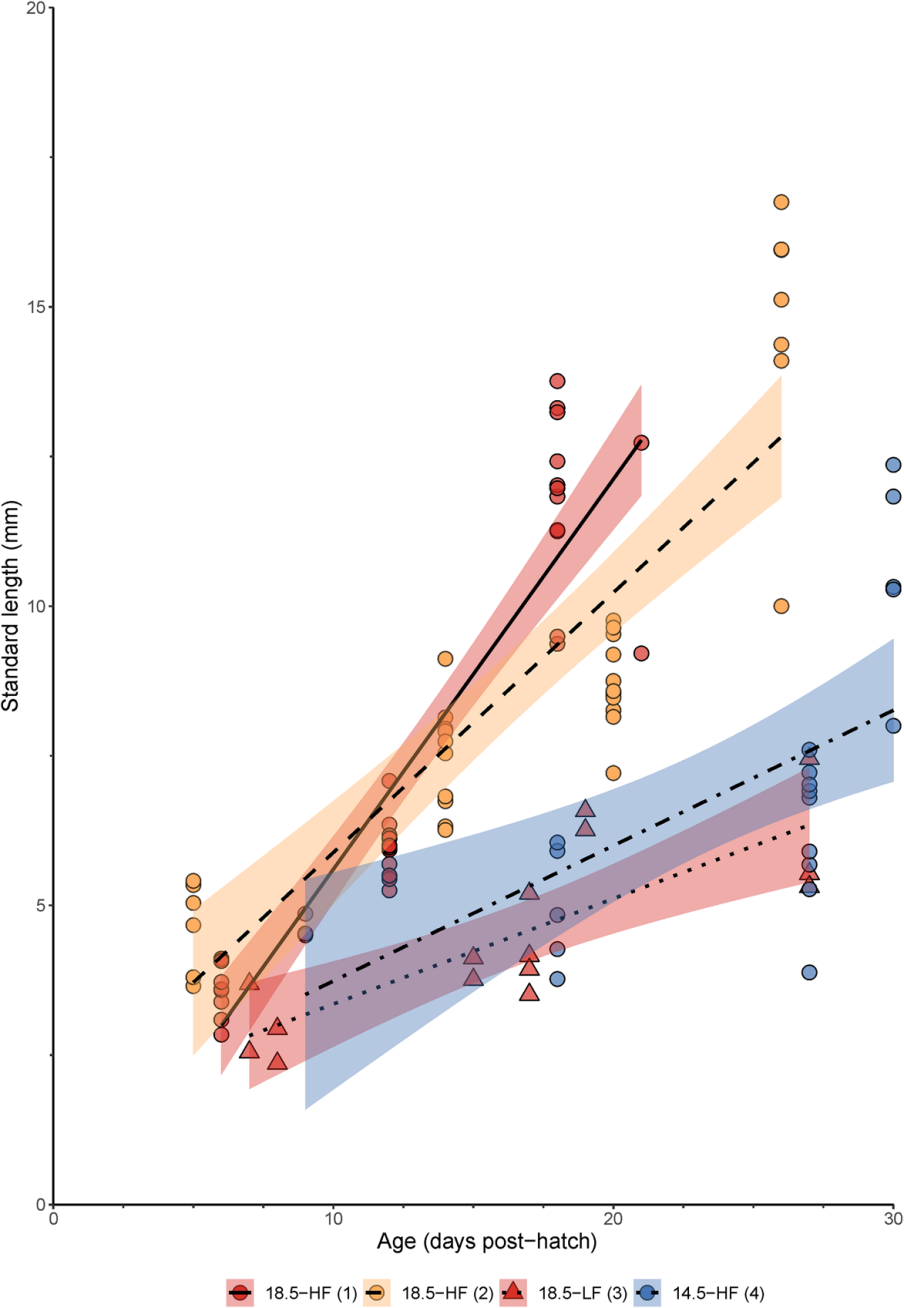
Relationship between standard length (SL, mm) and age (days post-hatch) in larvae of Peruvian anchovy, *Engraulis ringens*, reared in different conditions. 18.5-HF (1): 18.5 °C, high food, batch-1; 18.5-HF (2): 18.5 °C, high food, batch-2; 18.5-LF (3): 18.5 °C, low food, batch-3; 14.5-HF (4): 14.5 °C, high food, batch-4. Median slopes (Siegel estimator) and confidence intervals are reported in Table 1.

Peruvian anchovy larvae were reared at two temperatures (14.5 and 18.5 °C) and prey concentrations (ad libitum—high [HF] and low [LF]) from 6 to 30 days post-hatch (dph) to measure growth rate and examine the deposition of otolith increments (Fig. 1). Larval growth was affected by both temperature and prey concentration (Kruskal–Wallis, H (3) = 50.7, *P* < 0.005). Significant differences were observed in 18.5-HF between batch-1 and batch-2 (Pairwise Dunn’s test, *P* < 0.005), therefore, these batches were treated separately. Highest differences in median growth rates were observed between larvae reared at 18.5-HF (batch-1) and 18.5-LF (batch-3) (Pairwise Dunn’s test, *P* < 0.005), and between 18.5-HF (batch-1) and 14.5-HF (batch-4) (Pairwise Dunn’s test, *P* < 0.005) (Fig. 2).

Smaller differences were observed between 18.5-HF (batch-2) and 18.5-LF (batch-3) (Pairwise Dunn’s test, *P* < 0.05), while there was no significant difference in the median growth rate of larvae from 18.5-HF (batch-2) and 14.5-HF (batch-4) (Pairwise Dunn’s test, *P* = 1.00) and between 18.5-LF (batch-3) and 14.5-HF (batch-4) (Pairwise Dunn’s test, *P* = 0.42) (Table 1, Fig. 2). The median larval SL-at-age was also influenced by both variables (Table 1).

**Table 1:** Growth rate of *Engraulis ringens* reared at two temperatures (14.5 and 18.5 °C) and feeding (high food, HF and low food, LF) conditions.

Batch	Temperature (°C)	Treatment	Age (dph)	Growth rate (mm day^−1^)	CI 95%	*n*	Equation
1	18.5	HF	6–21	0.58 ± 0.12^a^	0.56 –0.71	35	SL = − 0.86 + 0.58 Age
2	18.5	HF	5–26	0.32 ± 0.09^b^	0.30–0.43	34	SL = 2.51 + 0.32 Age
3	18.5	LF	7–27	0.16 ± 0.05^c^	0.14–0.25	15	SL = 1.42 + 0.16 Age
4	14.5	HF	9–30	0.27 ± 0.28^b,c^	0.27–0.47	22	SL = − 0.025 + 0.27 Age

Values are expressed as median slopes ± median absolute deviation estimated using a Siegel's repeated medians estimator of median slopes. Different superscript letters indicate statistical differences. Median slopes for the standard length (SL, mm) and age (Age, days post-hatch) are reported, and slopes were statistically significant (*P* < 0.001). Confidence intervals (CI) and sample size (n) are also reported.

**Figure 3:**
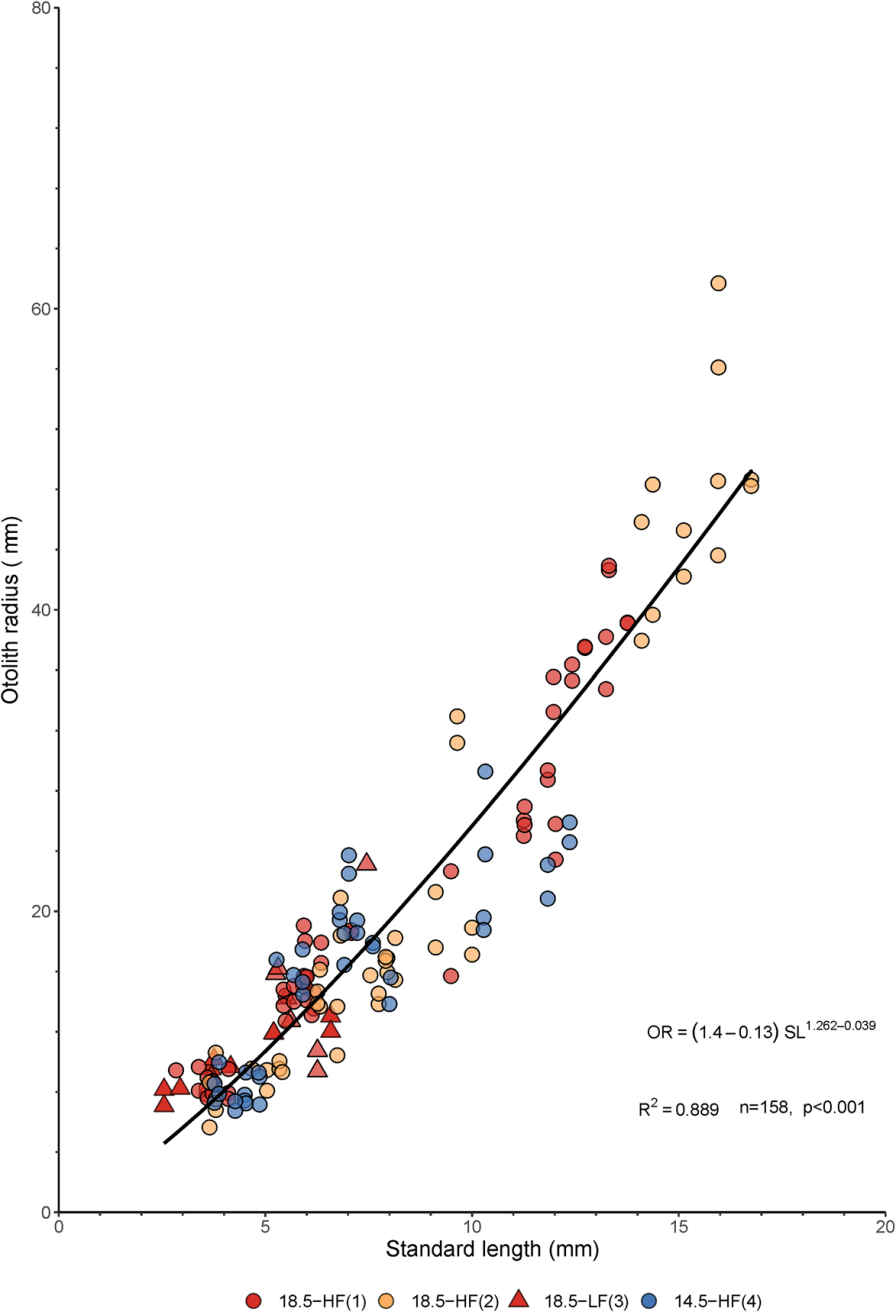
Relationship between otolith radius (OR, µm) and standard length (SL, mm) in larvae of Peruvian anchovy, *Engraulis ringens*, reared in different conditions. 18.5-HF (1): 18.5 °C, high food, batch-1; 18.5-HF (2): 18.5 °C, high food, batch-2; 18.5-LF (3): 18.5 °C, low food, batch-3; 14.5-HF (4): 14.5 °C, high food, batch-4. Regression equation provides estimates ± standard error.

**Figure 4:**
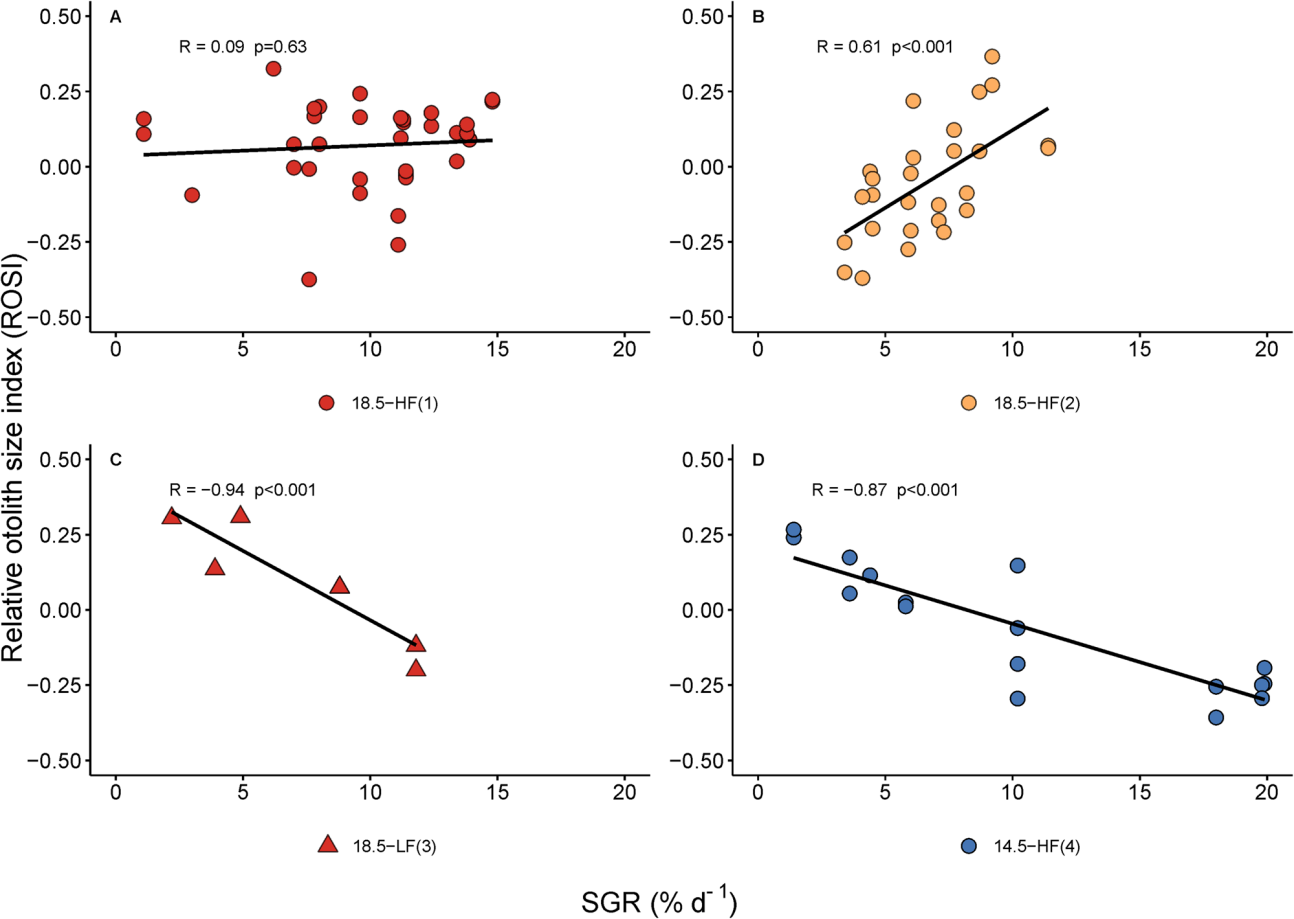
Relationship between relative otolith size index (ROSI) and specific growth rate (SGR, % d^−1^) in Peruvian anchovy larvae, *Engraulis ringens*, reared inr different conditions. (**A**) 18.5-HF (1):18.5 °C, high food, batch-1; 18.5-HF (2): 18.5 °C, high food, batch-2; (**B**) 18.5-LF (3): 18.5 °C, low food, batch-3; (**C**) 14.5-HF (4): 14.5 °C, high food, batch-4. The Pearson's correlation coefficient (R) is reported on top of each panel.

Otolith radius (OR) increased with standard length (SL) following a power function (r^2^ = 0.87, n = 158, *P* < 0.001) (Fig. 3). Values of the relative otolith size index (ROSI) were not statistically different among treatments (Kruskal–Wallis, H (3) = 9.11, *P* = 0.03, Pairwise Dunn’s test, *P* > 0.05), however, they were positively correlated with larval specific growth rate (SGR) in 18.5-HF batch-2 (R(25) = 0.61, *P* < 0. 0001), and negatively correlated in 18.5-LF and 14.5-HF (R(7) = − 0.94, *P* < 0.001; R(15) = -0.87, *P* < 0.001, respectively) (Fig. 4). The two variables (ROSI and SGR) were not correlated in the 18.5-HF batch-1(R(33) = 0.09, *P* = 0.63).

**Figure 5:**
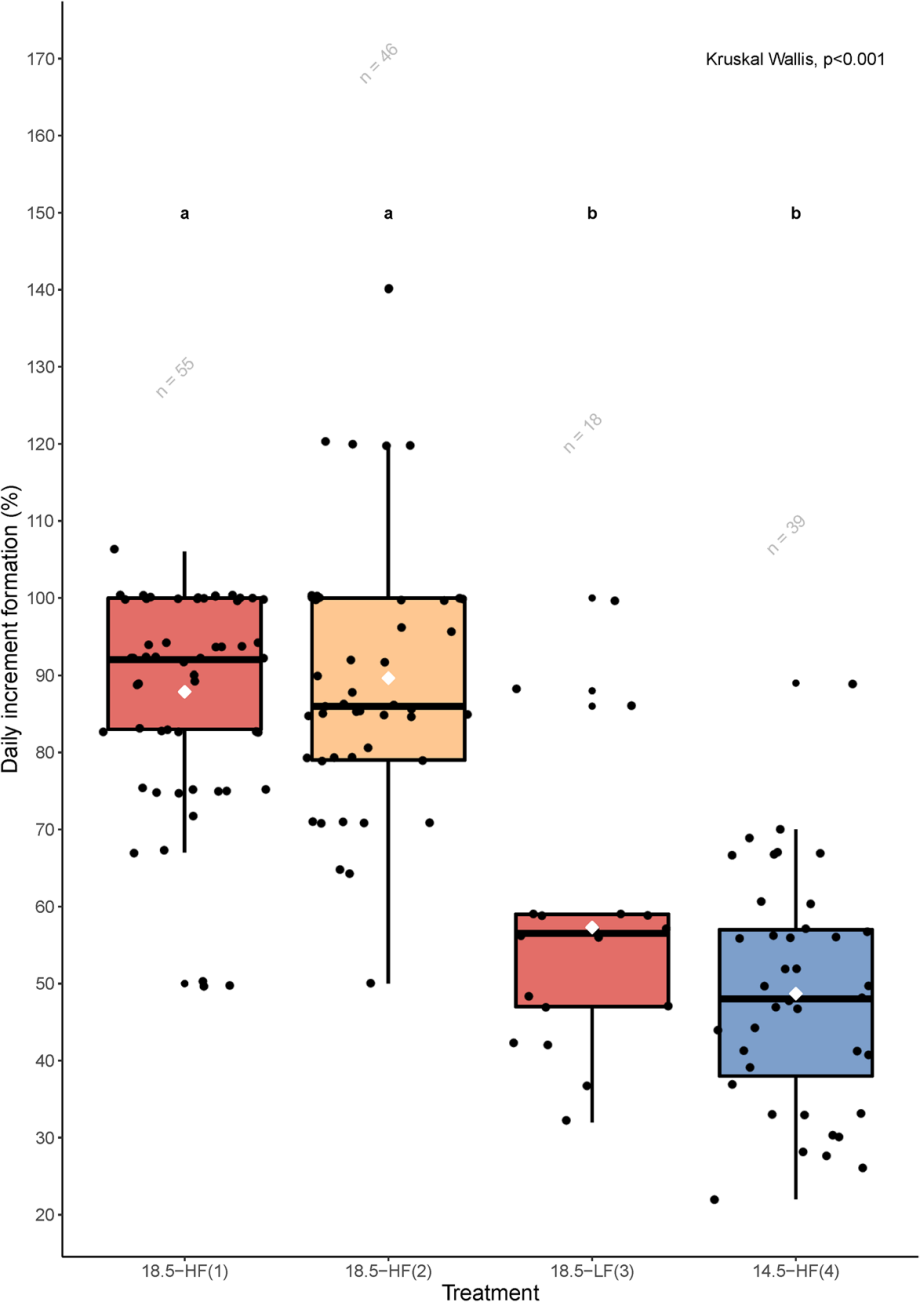
Percentage of daily otolith increment formation in larvae of the Peruvian anchovy, *Engraulis ringens,* reared in different conditions. 18.5-HF: 18.5 °C, high food, batch-1 and batch-2; 18.5-LF: 18.5 °C, low food, batch-3; 14.5-HF: 14.5 °C, high food, batch-4. Different superscript letters (**a**, **b**) indicate statistical differences (Pairwise Dunn's test, P < 0.001). Box plots show the 25th and 75th percentiles (lower and upper bounds of the box, respectively), the median (solid line), the 10th and 90th percentiles (whiskers), the mean (white dot) and outliers (black dots). Sample size is reported on top of each treatment.

**Figure 6:**
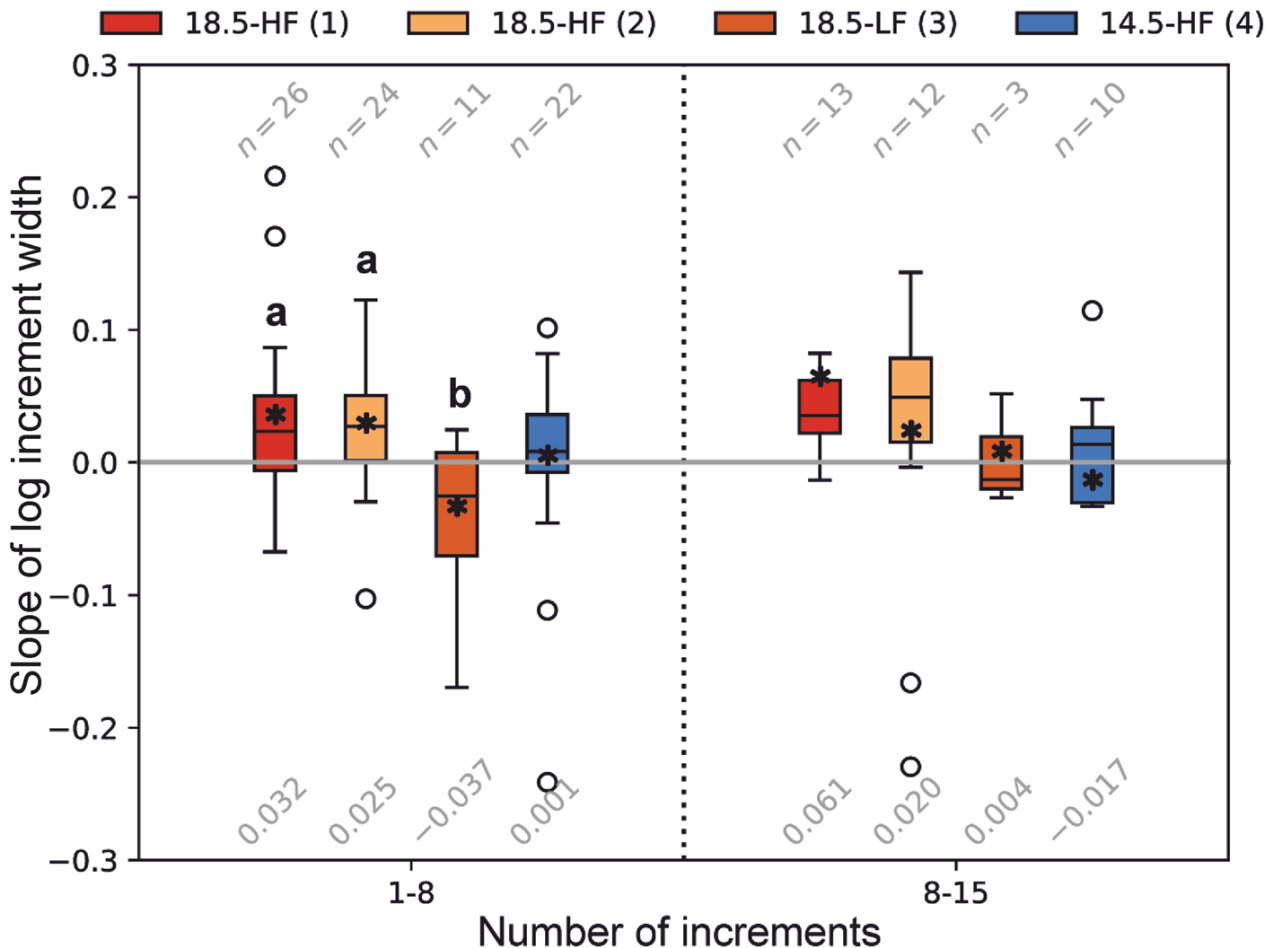
Slope of the log increment width versus increment number at two ranges of otolith increments numbers of Peruvian anchovy larvae, *Engraulis ringens,* reared in different conditions. 18.5-HF (1): 18.5 °C, high food, batch-1; 18.5-HF (2): 18.5 °C, high food, batch-2; 18.5-LF (3): 18.5 °C, low food, batch-3; 14.5-HF (4): 14.5 °C, high food, batch-4. Different superscript letters (**a**, **b**) indicate statistical differences (Pairwise Dunn's test, *P* < 0.05). Sample size is reported on top of each treatment and the respective means on the bottom.

A one-sample Wilcoxon signed-rank test on pooled data indicated that the median of the daily increments formation (DIF, %) was significantly different to 100 (V = 280.5, *P* < 0.001). A Kruskal–Wallis test (H (3) = 69.63, *P* < 0.001) revealed treatment-specific differences in the percentage of daily increments formed. Individuals from treatments 18.5-HF(1) and 18.5-HF(2) had a mean daily increment formation of 87.9% and 89.7%, respectively, which was statistically different to individuals from treatments 18.5-LF(3) and 14.5-HF(4) with a mean daily increment formation of 54.8% and 48.7%, respectively (Pairwise Dunn’s test, *P* < 0.001) (Fig. 5). Similar to larval growth rate, the slope of the log increment width versus increment numbers 1–8 was significantly different across treatments (Kruskal–Wallis, H (3) = 9.76, *P* < 0.020) (Fig. 6). The largest differences were observed between larvae in the HF and LF batches (18.5-HF batch-1 and 18.5-HF batch-2 versus 18.5-LF batch, Pairwise Dunn’s test, *P* = 0.029 and *P* = 0.027, respectively).

## Discussion

Peruvian anchovy larvae have rarely been studied under controlled laboratory conditions^3^. While this is surprising given that this species is responsible for the most valuable fishery worldwide, the arduousness of rearing small pelagic fish in captivity is well known. Due to this, fundamental information is lacking on the impact of temperature and prey availability on larval growth and otolith formation as well as other basic features of early development.

The temperatures used in this study (14.5 to 18.5 °C) characterize Peruvian coastal waters during Austral winter and Austral summer^29^. Furthermore, these seasons, (from August to March) coincide with the main spawning peak of Peruvian anchovy^30^. Moreover, the reproductive season occurs at the same time as the maximum upwelling activity^31^, such that a high abundance of Peruvian anchovy larvae tends to coincide with periods of relatively high biovolumes of phytoplankton and zooplankton in the HCS off Peru^7,32^.

The growth rates observed in our experiment (Table 1) are within the range of those reported for Peruvian anchovy larvae in the wild at slightly lower temperatures (12–16 °C, Table 2). Although recent studies reported a decline in the somatic growth rate of Peruvian anchovy at temperatures above 15 °C^33^, there are no growth records for wild-caught larvae at temperatures above 16 °C. This lack of knowledge may be because studies conducted so far have been carried out in Chilean coastal waters, where the sea temperature is colder than in Peruvian waters. As we observed that Peruvian anchovies grew faster at 18.5 °C (0.32 and 0.58 mm d^−1^) compared to 14.5 °C (0.27 mm d^−1^) (Tables 1 and 2), our results suggest, therefore, increased growth rates above those Chilean water temperatures. However, caution is needed when comparing these growth rates, as there are other factors beyond water temperature and prey concentration that will influence growth rate. For example, parental effects influenced the seasonal variability in growth rate of anchovy larvae in Chilean waters, where parents with higher energetic reserves at the beginning of the spawning season produced larvae with faster growth rates and higher survival compared with subsequent cohorts^34^.

**Table 2:** Compilation of growth rates ± standard deviation in wild-caught and laboratory-reared Peruvian anchovy (*Engraulis ringens*) larvae.

References	Temperature (°C)	Growth rate (mm day^−1^)	Larval size range (mm SL)	Source of larvae	Feeding conditions	Location
Present study	18.5	0.58 ± 0.13	3.6–11.0	Laboratory	High food	Peru
Present study	18.5	0.32 ± 0.09	4.7–14.6	Laboratory	High food	Peru
Present study	18.5	0.16 ± 0.05	3.1–6.1	Laboratory	Low food	Peru
Present study	14.5	0.27 ± 0.18	4.6–10.6	Laboratory	High food	Peru
Molina-Valdivia et al.^33^	12.0–16.0	0.21–1.82	2.9–23.2	Wild	In situ	Central Chile
Contreras et al.^34^	15.2	0.85 ± 0.02	2.5–16.8	Wild	In situ	Northern Chile
	14.2	0.50 ± 0.03	2.4–11.2			
Hernandez and Castro^35^	11.1–13.5	0.47	5.7–20.7	Wild	In situ	Central Chile
Castro and Hernandez^25^	11.0	0.68–0.79	3.7–26.1	Wild	In situ	Central Chile
Herrera et al.^36^	12.5	0.66–0.24	5.0–25.0	Wild	In situ	Central Chile

Growth rates from wild-caught larvae are estimated from otolith-based age determination, while laboratory-reared larvae are based on known-age.

More pronounced differences in growth rate between laboratory reared and wild-caught larvae have been reported for other anchovy species. For example, laboratory-reared European anchovy (*Engraulis encrasicolus*) larvae (15–20 dph) grew between 0.17 and 0.31 mm d^−1^ at temperatures that corresponded with increased larval foraging activity and growth^37^ while growth rates of wild-caught larvae were between 0.19 and 1.47 mm d^−1^ in the Bay of Biscay^38^ and between 0.40 and 0.94 in the Mediterranean^39^ and Aegean Sea^40^. Several reasons have been suggested to explain slower growth rates in the laboratory compared to the wild. First, size-selective mortality occurs in the wild, which tends to remove the smaller individuals increasing mean growth rates^41^. Second, rearing conditions in captivity may not be optimal in terms of prey diversity and quality compared to the wild, so it is very common that fish grow slower than in their natural environment^28^. Finally, our results suggest that, under certain conditions, growth rates of wild-caught larvae could be overestimated if otolith increments are not formed daily since age determination is done via otolith microstructure analysis. Thus, getting reliable estimates of optimal and critical thermal limits can help to identify stage-specific temperature threshold and thermal requirements that could be used for stock management^42,43^.

In the present study, almost a three-fold difference was observed between the median growth rates of larvae reared at 18.5 °C at high versus low prey concentrations. Since these ranges in temperatures and prey concentrations are those experienced by very young larvae in the field, our results support the importance of high prey concentrations for the growth of Peruvian anchovy larvae. However, ocean-climate fluctuations and seasonal primary production variability may lead to a mismatch between larvae and their prey, resulting in large recruitment variations in Peruvian anchovy in the HCS^9,44^. During warm periods, particularly in response to the ENSO of 1972-1973, phytoplankton and zooplankton abundances drastically decreased due to restricted upwelling conditions, reducing foraging opportunities for juveniles and adult anchovies^9^. Climate change scenarios (RCPs 4.5 to 8.5) project a moderate decrease in coastal upwelling strength and primary productivity and an increase in nearshore sea surface temperatures between 2 and 4.5 °C by 2100 in Peruvian waters^4,10^. These projections suggest likely poleward shifts of Peruvian anchovy populations, as spawning areas are associated with moderate to strong upwelling^45^.

The principle of age-determination via otolith microstructure analysis in cold-temperate fish is based on daily increment formation, where the number of bands correspond to the days in a year^46^. As suggested by the “growth-effect theory”, slow-growing larvae have larger otoliths compared to fast-growing larvae of the same age^47–49^. Nonetheless, under extreme conditions of starvation and low temperature, the daily deposition can be interrupted or altered, resulting in a 20 to 40%^27,50^ underestimation in the real age of larvae.

In our study, increments were deposited almost daily at the warmest temperature and high prey concentrations (18.5-HF batch-1 and batch-2). In addition, the constant photoperiod applied did not preclude the formation of daily increments. However, our study demonstrates that, even under high prey concentrations, 40% of the daily rings were not formed at a water temperature of 14.5 °C, producing a two-fold underestimation of age. This effect has been previously reported in larvae of several species of small pelagic fish such as Atlantic herring, *Clupea harengus*^26,51,52^, European anchovy^50^ as well as for adults and juveniles of Peruvian anchovy^27,53^. Similarly, daily increment formation was not observed in Japanese anchovy during winter when larvae experienced cold temperatures and exhibited slow rates of somatic growth^54^. The absence of daily increment formation has not only been related to slow rates of somatic growth but also to the resolution of the microscope. Light microscopy, the routine method used for examining otoliths readings in the larvae and later stages of marine fish, may lack the fine resolution necessary to detect very narrow increments formed during the first weeks after hatch. For example, in European sardine, *Sardina pilchardus*, very narrow increments formed during the first week post-hatch in well-fed larvae (3–30 dph) were detected by scanning electron microscopy but not by light microscopy^55,56^. On the other hand, larvae growing below 0.40 mm d^−1^ may not form daily increments, as reported for Atlantic herring, where increments between day 16 and 37 were not visible even using scanning electron microscopy^18^. Given that the growth rates at low temperature and prey concentrations in this study were around 0.20 mm d^−1^, it is plausible that daily increments were not formed, although this needs to be confirmed using higher-resolution microscopy. Our results demonstrated that when larvae were reared at temperatures of about 18.5 °C and at high prey concentrations (18.5-HF), the slope of the log increment width versus the number of daily increment formed was positive and with higher values compared to Peruvian anchovy larvae reared under low prey concentrations or at a temperature of 14.5 °C. In fact, under low prey concentrations (18.5-LF), the slope was negative when calculated across increments 1 to 8 and very low when calculated for increments 8 to15. When larvae were reared at temperature of about 14.5 °C (14.5-HF), the slope was similarly low in both 1 to 8 and 8 to 15 increment ranges. Our results, therefore, support the hypothesis that under conditions of low prey concentrations or low temperature, otolith increments either do not form on a daily basis, or form but are too narrow to discern with standard light microscopy.

Even though the relative otolith size index (ROSI) was not statistically different among treatments, the relative otolith size index (ROSI) and larval specific growth rate (SGR) were positively correlated at the warmer temperature and high prey concentrations, suggesting a positive relationship between somatic growth and otolith growth under favourable conditions.

Unfortunately, due to time constrains caused by the global pandemic, it was not possible to replicate some of the experiments. Further data collection is required to determine the mechanism(s) by which unfavorable growth conditions (e.g. colder temperature and/or low prey concentration) influence otolith formation.

The otolith analysis, together with biomass and oceanographic data (e.g., temperature, primary production), allows the back-calculation of age and growth commonly used to determine the annual productivity of *E. ringens* stocks^57^. However, for a better assessment of wild populations, an adjustment should be applied by considering the original water temperature populated by new recruits. In case of otolith microstructure analysis, the resolution of the microscope used for age estimation should also be taken into consideration.

In the present study, the growth rate obtained at warmer temperature and high prey density was commensurate to that reported for both laboratory and field-collected larvae, providing also the most realistic approximation to the real-age of larvae. At a temperature of about 18.5 °C and high prey concentrations, otoliths represent a valuable proxy for somatic growth and age estimation of *E. ringens* larvae, as the increment width increased with subsequent daily increments, which was detectable with traditional light microscopy. At low prey concentrations or low temperature (14.5 °C), significant discrepancies were found between the expected and observed number of daily increments, leading to underestimates of age and bias when otolith derived ages are used to augment stock assessment surveys. This is the first study reporting the effect of temperature and prey concentrations in laboratory-reared Peruvian anchovy larvae. It contributes to the growing body of evidence highlighting the prominent importance of environmental variables in the assessment of Peruvian anchovy populations in the Humboldt Current System under current and future climate change scenarios.

## Materials and methods

### Rearing system

Adult Peruvian anchovies were captured off the coast of Lima, Peru, in July 2019 by commercial artisanal purse-seiners and transferred to the aquaculture facilities of the Peruvian Marine Institute (IMARPE). Broodstock collection, acclimation, rearing and eggs collection was performed following the protocol described by Rioual et al.^28^. Briefly, anchovy adults were maintained in three cylindrical, dark blue, fiberglass tanks of 2000 L each, connected to a recirculating aquaculture system (RAS, total recirculation flow rate 4000 L h^−1^, allowed total volume exchange every 30 min). The rearing system consisted of a mechanical sand filter supplied with natural seawater pumped from a 20,000 L reservoir, reverse osmosis filters (10, 5 and 1 µm cartridge) and a mechanical biofilter.

The water temperature remained constant at 18 °C and the light regime was adjusted to 13 h of light and 11 h of dark. Constant aeration was provided. Fish were fed manually 3 × d^−1^ with 2-mm commercial pellets (slow-sinking type, Otohime EP2, 48% protein and 14% lipids) previously enriched with fish oil and multivitamin supplement (vitamins A, E and C, Hematec® TQC, 0.05% of food weight). After two months of acclimation to laboratory conditions, fish spawned naturally within tanks. At the beginning of the spawning season, mean ± SD body length of males and females was 13.3 ± 0.5 and 14.2 ± 0.5 cm, respectively. Eggs from four separate spawning events (mean ± SD time that elapsed between each successive spawning event was 12 ± 5 days) were collected in a 100-µm fine mesh sieve placed at the tank outflow, transferred to 4.5-L rectangular tanks and incubated at two temperatures, 14.5 °C (cold treatment) and 18.5 °C (warm treatment). The gentle acclimation of eggs in the cold treatment started at a room temperature of about 19.0 ± 0.5 °C and cooled by a chiller unit until reaching the designated target temperature within 9 h (0.5 °C h^−1^). Dead eggs were removed daily by siphoning the bottom.

After hatching, larvae from each spawning event were transferred to individual circular tanks with a maximum total volume of 100 L filled with 60 L of filtered seawater sterilized with a 36W UV lamp and gentle aeration. A daily water exchange of 5% was performed by siphoning the tank bottom. Before the yolk sac was completely absorbed (2 dph), larvae from two of the warm tanks received high food concentrations (18.5-HF, batch-1 and batch-2); another warm tank received low food concentrations (18.5-LF, batch-3) and one cold tank was supplied with high food concentrations (14.5-HF, batch-4) (Fig. 1). In the high food treatments (HF), larval feeding was initiated by providing the dinoflagellate *Akashiwo sanguinea* (100 cell ml^−1^ day^−1^) from 1 to 6 dph, followed by the rotifer *Brachionus plicatilis* (20 rotifers ml^−1^ day^−1^) from 3 to 26 dph and *Artemia sp.* nauplii (2 nauplii ml^−1^ day^−1^) from 25 dph until the end of the experiment at 30 dph. Larvae in the low food treatment (LF) were provided with *A. sanguinea* (18 ± 9 cell ml^−1^ day^−1^) from dph 1 to 11 and *A. sanguinea* + *B. plicatilis* (8 ± 4 rotifers ml^−1^ day^−1^) from 11 to 30 dph. All treatments received *Tetraselmis sp.* microalgae (100,000 cell ml ^−1^) from hatch until 26 dph. For the first 23 dph, all tanks received constant light which was shifted to a 13:11 h light/dark (L:D) regime afterwards. Oxygen, temperature and salinity were recorded daily with a multiparameter portable meter (WTW multi 3430) fitted with an optical Imaging Development System (IDS) dissolved oxygen sensor (WTW, FDO 925, precision 0.01 mg O_2_ l^−1^ and 0.1 °C).

### Growth sampling

Fish were sampled weekly from a random selection of between 2 and 10 larvae from each rearing tank. Larvae were anesthetized with 0.1 g L^−1^ of tricaine methanesulfonate (MS-222, Syndel, Qualicum Beach, BC Canada) and then euthanized with an overdose of MS-222 before preservation in 96% ethanol. Digital pictures were taken under stereomicroscope for length measurements (± 0.1 mm, Leica MZ 16, Wetzlar, Germany). Standard length (SL) was measured from the tip of the snout to the tip of the notochord in pre-flexion larvae, and from the tip of the snout to the posterior end of the hypural plate in post-flexion larvae. No correction for ethanol preservation was applied^58^.

### Otolith analysis

The otolith microstructure analysis was carried out at the University of Hamburg, Institute of Marine Ecosystem and Fishery Science (IMF). A total of 158 otoliths were extracted from 81 larvae. The extraction was performed by dissecting the larva under a stereomicroscope equipped with a polarized filter. Both sagittal and lapillar otoliths were removed from each side of the saccular chamber and transferred to a clean microscope slide within a drop of glue. The microstructure analysis was performed only in sagitta otoliths with an Olympus Binocular Microscope connected to a high-resolution camera (Leica DC 300), providing a theoretical resolution of 0.078 μm pixel^−1^. A multi-frame image was created from several images captured at 1000 × magnification at different focal planes to improve the identification of the increments. The cumulative otolith radius (OR, µm), hatch check (HC, µm), number of increments and the widths between successive increments were measured along a transect at a 90° angle from the shortest otolith axis. To interpret the otolith growth increment, the Group Band Reading (GBR) method was used and each micro-increment that included a light and dark band was counted as a daily increment^59^. A second reader analysed the otoliths, and differences in the number of increments between both readers were generally ± 0–3 increment. Only data from the first reader were used for the statistical analyses.

### Statistical analysis

Growth analysis (based on SL) was performed in 121 larvae between 5 and 30 dph. Growth rate (mm day^−1^) was calculated from length-at-age and determined and compared using the nonparametric Siegel's repeated medians estimator for slopes regressions and a 95% confidence interval (CI) was calculated by applying the “confint.mblm” function of the “mblm” R package to each of the four regressions. A Kruskal–Wallis-Test was applied to the full sample set (106 individual fish) and treatment differences tested by a post-hoc Dunn test with Bonferroni adjustment. The relationship between SL and OR was regressed with a power function using a non-linear least square method. SL and OR data were then log-transformed and residuals measured as proxies of relative otolith size index (ROSI)^34^. Positive relative otolith size index (ROSI) values would correspond to fish that have larger otolith radius than expected for an “average” larva at the same length. To analyse the correlation between somatic growth and otolith growth, specific growth rate (SGR, % d^−1^) was plotted against relative otolith size index (ROSI) values, and the association between the two variables was quantified with the Pearson's correlation coefficient. The SGR was calculated as following:

$$SGR = \frac{{\left( {\ln Lf - \ln Li} \right)}}{t}*100$$

where $\ln$ *Lf* is the natural log of final length; $\ln$ *Li*, the natural log of initial length, and *t,* the elapsed time in days. In fish from each treatment and batch, the proportion of number of daily increments formed (DIF, %) was calculated as:

$$DIF=\frac{{I}_{obs}}{{I}_{exp}}*100$$

where I_*obs*_ is the observed number of daily increments, and I_*exp*_, the number of increments expected from the known age of the larva. A one-sample Wilcoxon signed-rank test with continuity correction was computed on pooled data to assess whether the observed daily increments formed (DIF) were different to 100%, while treatment differences with a Kruskal–Wallis-Test and post-hoc Dunn test with Bonferroni adjustment.

The slope of log increment width versus increment number

$$Slope\;of\;log\;increment\;width = \frac{{\Delta log\;increment\;width}}{{\Delta increment\;number}}$$

was computed by linear fitting of the two variables for each individual larva and over two ranges of numbers of increments (1–8 and 8–15). The slope values for individual larvae were pooled and visualized using a boxplot for each of the two ranges of numbers of increments (1–8 and 8–15) and each treatment. Treatment-specific differences were tested with Kruskal–Wallis-Test and post-hoc Dunn test with Bonferroni adjustment for each range of number of increments (1–8 and 8–15). All analyses were performed using R^60^ and Phyton.

### Ethical approval

All experimental procedures complied with the Animal Welfare Legislation approved by the Government of Peru (Law N_ 30407). The study was performed in strict accordance with the Guidelines of the European Union Council (Directive 2010/63/EU) under a personal licence to C.O. issued by the Spanish General Directorate of Forestry Management and Ministry of Rural Environment. All methods are reported in compliance with ARRIVE 2.0 guidelines^61^.

## Data Availability

The datasets analysed during the current study are available from the corresponding author on reasonable request.
